# Intratumoural microbiome can predict the prognosis of hepatocellular carcinoma after surgery

**DOI:** 10.1002/ctm2.1331

**Published:** 2023-07-18

**Authors:** Lejia Sun, Xindi Ke, Ai Guan, Bao Jin, Jiangming Qu, Yinhan Wang, Xiang Xu, Changcan Li, Hang Sun, Hengyi Xu, Gang Xu, Xinting Sang, Yifei Feng, Yueming Sun, Huayu Yang, Yilei Mao

**Affiliations:** ^1^ Department of Liver Surgery, Peking Union Medical College (PUMC) Hospital PUMC & Chinese Academy of Medical Sciences Beijing China; ^2^ Department of General Surgery The First Affiliated Hospital of Nanjing Medical University Nanjing China; ^3^ The First School of Clinical Medicine Nanjing Medical University Nanjing China; ^4^ Colorectal Institute of Nanjing Medical University Nanjing China; ^5^ Eight‐year MD Program Peking Union Medical College (PUMC) & Chinese Academy of Medical Sciences Beijing China; ^6^ Liver Transplant Center, Organ Transplant Center West China Hospital of Sichuan University Chengdu China; ^7^ Laboratory of Liver Transplantation, Key Laboratory of Transplant Engineering and Immunology, NHC West China Hospital of Sichuan University Chengdu China

**Keywords:** hepatocellular carcinoma, hepatotype, intratumoural microbiome, prognosis

## Abstract

**Background:**

The dismal prognosis of hepatocellular carcinoma (HCC) is closely associated with characteristics of the tumour microenvironment (TME). Recent studies have confirmed the presence and potential influence of the microbiome in TME on cancer progression. Elucidating the relationship between microbes in the TME and cancer could provide valuable insights into novel diagnostic markers and therapeutic strategies for HCC and thus warrants a closer investigation of the role of intratumoural microbiome in the HCC TME.

**Methods:**

We determined the presence of intratumoural microbiome using fluorescence in situ hybridisation, and explored the microbial community profiles in the HCC TME in paired tumour and adjacent normal tissues using 16S rDNA sequencing. Microbial signatures were characterised in the paired group, and their correlation with clinical characteristics was further investigated. We clustered the microbial signatures of tumour tissues by hepatotypes, and further analysis was performed to elucidate the independent prognostic value of the hepatotypes.

**Results:**

This study revealed that microbial profiles and community networks differed notably between tumours and adjacent normal tissues. Proteobacteria and Actinobacteria were the most abundant phyla in the HCC TME. The TME microbial profiles also revealed heterogeneities between individuals and between multiple tumour lesions. Clustering of the microbial profiles into two hepatotypes revealed different microbial network patterns. Additionally, the hepatotypes were revealed to be independent prognostic factors in patients with resected HCC.

**Conclusions:**

Our study illuminates the microbial profiles in the TME of HCC and presents the hepatotype as a potential independent biomarker for the prognostic prediction of HCC after surgery.

## Graphical abstract

- HCC tumours possess a distinct microbiome in comparison to the adjacent tissues.
- The intratumoural microbiome of HCC displays heterogeneity.
- The hepatotype is developed based on clustering analysis of the microbial profiles.
- The intratumoural microbiome predicts HCC prognosis.

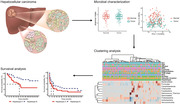

## 1 INTRODUCTION

Liver cancer ranks sixth among the most frequently diagnosed cancers and third among the leading cause of cancer‐related deaths globally.^1^ Hepatocellular carcinoma (HCC) accounts for over 80% of all primary liver cancers.^2^, ^3^ An HCC tumour comprises a complex tumour microenvironment (TME), containing cellular, chemical and physical components. TME plays a significant role in clinical outcomes and response to therapy for HCC.^4^ The composition of the TME is widely recognised as the key determinant of solid tumours, influencing their development, growth and progression.^5^ Elucidating the relationship between the TME and cancer could provide valuable insights into the identification of novel diagnostic markers and development of therapeutic strategies for HCC.

Recent studies have detected the presence of microbiota in the TME,^6^, ^7^, ^8^ and report that microbes inhabiting mucosal sites may be incorporated into the TME of aerodigestive tract malignancies. Intratumoural microbes may affect cancer development and progression in several ways.^9^, ^10^ Riquelme et al. revealed that the microbiome composition of pancreatic adenocarcinoma affected the patients’ immune response and disease progression.^7^ The cancer microbiota also reportedly influences cancer pathology by modulating tumourigenesis,^11^ cancer progression and chemotherapy resistance.^12^ Thus, the microbiota is drawing increasing attention as a critical factor in the TME. Elucidating the complex interplay between the microbes, TME and cancer cells can offer valuable insights into potential cancer treatments and help evaluate the effectiveness of existing treatments at the individual patient level.^6^, ^7^, ^13^

Recently, there have been increasing research efforts to investigate the intratumoural microbiome of HCC. Komiyam et al. utilised 16S rDNA sequencing to characterise the microbiota profiles in both primary and metastatic liver cancer tissues.^14^ Subsequently, two studies validated the presence of an intratumoural microbiome in HCC, and further linked the microbiome spectrum to patient clinical features.^15^, ^16^ Additionally, Liu et al. revealed that patients with HCC possessed a dysbiotic microbiota that triggered hepatic stellate cell activation senescence, thereby promoting hepatocarcinogenesis.^17^ Moreover, a multiomics investigation discovered that the intratumoural microbiome was closely related to metabolites and genetic alterations.^18^ Despite these developments, the clinical significance of the intratumoural microbiome in HCC is still poorly understood. While initial researches found a connection between the microbiome in liver cancer and patients' survival,^19^, ^20^ it is likely that their results were undermined by inadequate quality control or a limited sample size. Therefore, it is imperative to further investigate the profiles of the HCC tumour microbiome and clarify its clinical significance and prognostic value.

In this study, we primarily focused on exploring the microbiota as a component of the HCC TME. We performed paired tumour and normal tissue analyses to account for the microbial heterogeneity between them. We developed hepatotypes of microbial profiles in the HCC TME and analysed the patterns hoping to elucidate the interactions between the intratumoural microbiome and the clinical characteristics and prognosis of HCC. This study thus aimed to uncover the underlying critical relationship between the microbiome in the HCC TME and clinical prognosis.

## 2 MATERIALS AND METHODS

Participant tissue samples were retrospectively collected from the Peking Union Medical College Hospital (PUMCH). Written informed consent was obtained from all participants.

### 2.1 Sample collection

In total, 115 patients with HCC who underwent hepatectomy at PUMCH were enrolled. The median follow‐up duration was 79 months. The inclusion criteria were as follows: (1) evidence of histologically confirmed HCC, (2) presence of surgically resectable HCC, and (3) clinical data available upon first diagnosis. Patients receiving preoperative treatment and those with a history of other malignancies were excluded. Ninety‐one patients satisfied the inclusion criteria and passed the exclusion criteria required to proceed to the next stages of analyses. Paired liver tumour and paratumour tissues were collected from 78 patients. Only the tumour tissues were obtained from the remaining 13 patients. Eighteen tumour tissues were obtained from multiple lesions in eight cases.

All samples were obtained aseptically in the operating room, and subsequently cryopreserved and stored at −80°C storage for preservation, all within 30 min from the time of sample collection. Four cryovials of sterile saline that was kept exposed in the surgery room during sample collection were used as negative controls to eliminate the effect of potential contamination from the environment and subsequent operations.

### 2.2 DNA extraction and 16S rDNA sequencing

Microbial DNA was isolated from the samples using the Qiagen DNA Microbiome Kit (Tiangen) and quantified using a Nanodrop. The quality of the extracted DNA was evaluated using 1.2% agarose gel electrophoresis. Polymerase chain reaction (PCR) that targets the V3–V4 region of the 16S rRNA gene was performed using the following primers: forward primer, 5′‐ACTCCTACGGGAGGCAGCA‐3′; and reverse primer, 5′‐GGACTACHVGGGTWTCTAAT‐3′. Magnetic beads were used to purify and recover the PCR products, and the recovered products were subjected to fluorescence quantification. The sequencing libraries were prepared using the Illumina TruSeq Nano DNA LT Library Prep Kit. After obtaining the enriched products from the library, the library was subjected to final segment selection and purification using 2% agarose gel electrophoresis. Each qualified onboard sequencing library was gradient‐diluted and mixed according to the required ratio, and onboard sequencing was performed. Paired‐end amplicon sequencing was performed using the Illumina Nova Seq platform.

### 2.3 Sequencing data processing

After obtaining the raw sequencing data in FASTQ format, the primers and barcode sequences were removed, and quality control and filtering were completed. Next, the EasyAmplicon^21^ pipeline was used to analyse the data on usearch and vsearch software. Sequences that were less than 200 bp long were truncated and those with frequencies less than eight were removed to reduce sequence redundancy. Chimeras were identified using the Ribosomal Database Project (RDP) database and removed. The data were further denoised using the ‘Denoise’ algorithm in vsearch, and the filtered high‐quality sequences were assigned to amplicon sequence variants (ASV) at a cut of 97% sequence similarities. Species annotation was performed using the RDP database, and plastids were removed.

To reduce potential contaminations introduced by the environment and reagents, the following decontamination measures were implemented during the analysis: (1) ASVs of contamination were identified based on the ‘decontam’ algorithm^22^; (2) ASVs with a relative abundance of more than 0.5% in the control samples were removed; and (3) ASVs that appeared in less than 10% of the tissue sample were also removed to avoid contingency.

To demonstrate that the retrospective collection of samples during a long period does not compromise the reliability, we conducted 16S rDNA sequencing in three previously collected pairs of tumour and adjacent tissues and three pairs of samples prospectively collected. After the removal of contaminations, we compared the retrospectively collected samples with the prospective samples in terms of α‐diversity, β‐diversity, main composition at the phylum level and differential taxa at the ASV level (Figure S1). Both the α‐ diversity and β‐diversity showed no significant differences between the prospective and retrospective groups (Figure S1A and B). No notable differences were observed in the relative abundance of the main phyla (Figure S1C). In 206 ASVs with a relative abundance higher than 0.05%, only two ASVs and four ASVs were depleted and enriched in the prospective samples (Figure S1D). The six differential ASVs between the retrospective and prospective samples accounted for only <2.6% of all the sequences. These results indicated that the sample collection during a long period did not impact the results of our study with adequate decontamination procedures.

### 2.4 Microbiome analysis

The decontaminated ASV table was resampled to a minimum number of sequences form each sample before downstream analysis, including microbial diversity and composition. The α‐diversity was calculated using the R package ‘vegan’^23^ and displayed as Shannon indices. The β‐diversity was analysed via principal coordinate analysis (PCoA) based on Manhattan distance and partial least squares discrimination analysis (PLS‐DA). Microbial differences between groups were analysed using the R package ‘edgeR’^24^ and the linear discriminant analysis effect size (LEfSe) algorithm (http://huttenhower.sph.harvard.edu/lefse/).^25^ Hierarchical clustering was performed and visualised using MicrobiomeAnalyst (https://www.microbiomeanalyst.ca/).^26^ Hepatotypes were classified according to the hierarchical clustering of tumour samples via the Ward algorithm based on the Euclidean distance. Microbial network analysis and visualisation were performed using the R package ‘ggClusterNet’.^27^ PICRUSt^28^ was used for functional prediction of microbiomes based on the Greengene and Kyoto Encyclopedia of Genes and Genomes (KEGG) databases. Differential pathways were displayed via STAMP software.^29^ The effects of hepatotypes on recurrence‐free survival (RFS) and overall survival (OS) were investigated using Kaplan–Meier survival curves and Cox regression. Both the univariate and multivariate regression analyses included the hepatotype and traditional prognostic factors for HCC. Two‐tailed *p* values < .05 were considered statistically significant.

### 2.5 Fluorescence in situ hybridisation (FISH) and immunofluorescence

Fresh frozen tissues were fixed in 4% paraformaldehyde, embedded in optimal cutting temperature compound and then sectioned into slides. The slides were incubated in the prehybridisation solution for 1 h at 37°C. Next, the hybridisation solution containing the Cyanine 3‐labelled FISH probe EUB338 (5′‐GCT GCC TCC CGT AGG AGT‐3′), which is specific to bacteria 16S rRNA, was added. The samples were incubated overnight in a humid environment at 42°C. Immunofluorescence was conducted after FISH. The first step involved washing the slides with 0.1 M Tris‐HCl, subsequent to which they were permeabilised using 0.3% Triton X to facilitate access of antibodies to the relevant epitopes. For optimal specificity and sensitivity, the slides were then blocked with 2% foetal bovine serum for 30 min at ambient temperature. In the next step, the slides were exposed to primary antibodies (anti‐CD8 and anti‐CD68) overnight at 4°C, followed by fluorophore‐conjugated secondary antibodies for 1 h at ambient temperature. For the purpose of nucleic acid counterstaining, 4′,6‐Diamidino‐2‐phenylindole (DAPI) was employed at ambient temperature. The stained slides were mounted in a dark environment in DAPI antifade solution, and washed before being photographed under a fluorescence microscope (Nikon A1, Tokyo, Japan).

## 3 RESULTS

### 3.1 Population characteristics

Table 1 shows the clinical details of the included subsequent cases. Samples were collected from 91 patients who had undergone inclusion/exclusion procedures. The median age was 55 (35−83), and 83 patients (91.2%) were male. Alcohol intake was reported in 34 (37.4%) patients, HBV infection was observed in 73 (80.2%) patients, and type 2 diabetes mellitus was present in 15 (16.5%) patients. Sixty‐two (68.1%) patients were diagnosed with cirrhosis; among them, only five progressed to Child‐Pugh class B, while the other 57 patients were at Child‐Pugh class A. All patients achieved a negative surgical margin. The number of patients with TNM stages I, II and III were 45 (49.4%), 19 (20.9%) and 27 (29.7%), respectively. None of the participants developed lymph node or distant metastases at the time of surgery. Tumour differentiation was high (30.0%), medium (48.3%) or poor (20.9%).

**TABLE 1:** Clinical characteristics of patients.

Characteristics	
Age (year)	58 (35−83)
Gender (male)	83 (91.2%)
Alcohol intake (yes)	34 (37.4%)
HBV infection (yes)	73 (80.2%)
HCV infection (yes)	4 (4.4%)
Type 2 diabetes (yes)	15 (16.5%)
Body mass index (>24 kg/m^2^)	43 (52.4%)^*^
Cirrhosis (yes)	62 (68.1%)
Tumour differentiation	
Well	28 (30.8%)
Medium	44 (48.3%)
Poor	19 (20.9%)
TNM stage	
I	45 (49.4%)
II	19 (20.9%)
III	27 (29.7%)
Tumour size (> 5 cm)	53 (58.2%)
Tumour number (multiple)	31 (34.1%)
Macrovascular invasion (Yes)	20 (22.0%)

* Body mass index of 9 participants were unknown.

### 3.2 The presence of microbiome in hepatocellular carcinoma

**FIGURE 1:**
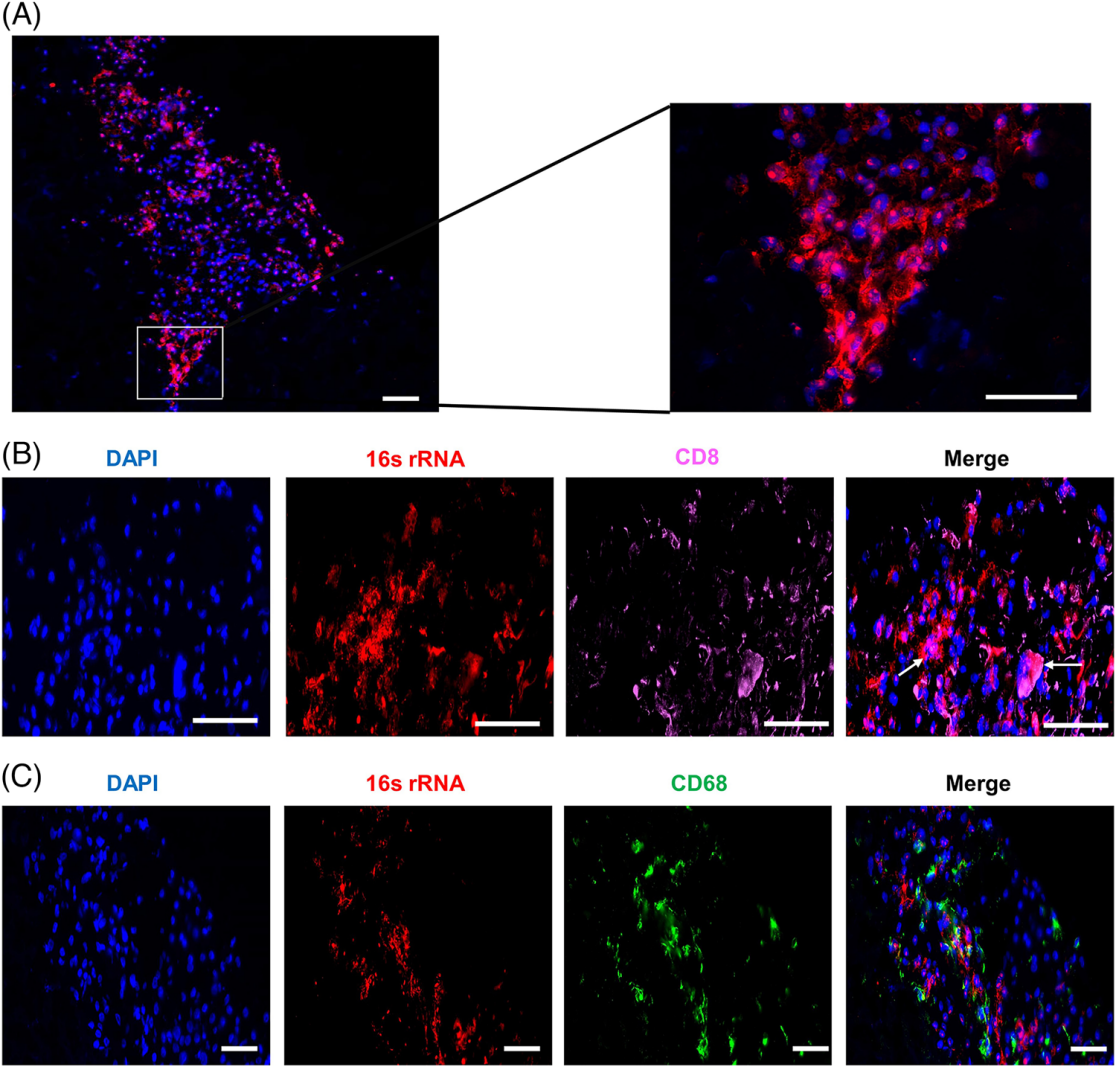
Presence of intratumoural microbiome in hepatocellular carcinoma and its costaining with immune cells. (A) Fluorescence in situ hybridisation (FISH) analysis of bacterial 16S rRNA in a tumour sample stained with EUB388 probe (red) and DAPI (blue). (B, C) Costaining of bacterial 16S rRNA with immune cells. CD8+ T cells are marked by pink (B), and macrophages are marked by green (C). The arrows indicate signals of intratumoural bacteria within CD8+ cells. Scale bar = 100 μm.

To reveal the presence of a microbiome in HCC, we performed FISH in a tumour sample using the EUB388 probe. The presence of bacteria in hepatocellular carcinoma was confirmed by the detection of FISH signals (Figure 1A). Additionally, the microbial distribution exhibited a certain degree of heterogeneity. We further explored the relationship between microbial presence and immune cells via FISH and immunofluorescence costaining. Some FISH signals were present in CD8+ cells, indicating the presence of bacteria within CD8+ cells (Figure 1B). The CD68+ macrophages tended to be distributed in areas where the intratumoural microbiome was more enriched (Figure 1C), suggesting that the intratumoural microbiome might influence the infiltration of macrophages in HCC.

### 3.3 Different microbial profiles in tumours and adjacent normal tissues

**FIGURE 2:**
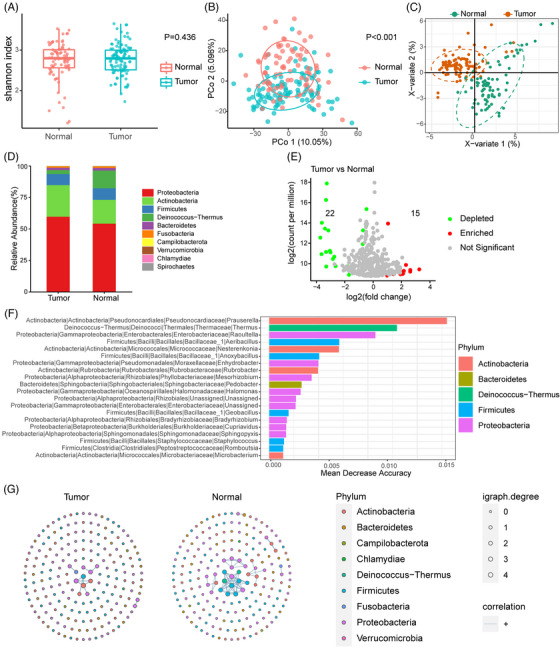
Characterisation of the microbiome in hepatocellular carcinoma (HCC) tumour and adjacent normal tissues. (A) Boxplot of Shannon index in the tumour and adjacent normal tissues. (B) Principal coordinates analysis (PCoA) based on Manhattan distance of the tumour and adjacent normal tissues. (C) Partial least squares discriminant analysis (PLS‐DA) of the tumour and adjacent normal tissues. (D) Bar plots of microbial composition at the phylum level in tumour and normal tissues. (E) Volcano plot of differential amplicon sequence variants (ASVs) between tumour and normal groups. ASVs significantly depleted in tumour tissues are shown in green; those significantly enriched in tumour tissues are shown in red (|log2 fold‐change| > 1, *p* < .05, and FDR < 0.2). (F) Bar plot of 20 genera with the top ability to discriminate tumour from normal tissues computed from a random forest model. The phyla are indicated by different colours. (G) Microbial networks in tumour and normal tissues. Each dot represents one ASV, and only 200 ASVs with top abundance are shown. The size of each dot represents igraph degree, which reflects the number of significant correlations between the corresponding ASV with the others. Blue lines indicate positive correlations.

Tumourous and adjacent samples from 91 patients with HCC were examined. The Shannon index of α‐diversity showed no significant differences between the tumours and adjacent tissues (*p* = .436, Figure 2A). The β‐diversity, which was calculated using Manhattan distance, revealed a significant difference between the tumour and normal groups (*p* < .001, Figure 2B). Additionally, PLS‐DA also revealed diversity between the two groups (Figure 2C). On average, four bacterial phyla (Proteobacteria, Actinobacteria, Firmicutes and Deinococcus‐Thermus) accounted for up to 90% of the sequences. These four phyla were dominant both in the tumour and adjacent normal tissues (Figure 2D). Actinobacteria was notably overrepresented in tumour tissues, whereas Deinococcus‐Thermus was significantly overrepresented in normal tissues (Figure S2).

Using edgeR, differential analysis was performed between the tumour and normal tissues to identify ASVs that were significantly dysregulated. The analysis showed that 15 ASVs were upregulated and 22 were downregulated in the tumour tissues (Figure 2E). The LEfSe method was used to identify taxa that could help differentiate tumours from normal tissues. Taxa of Actinobacteria, Proteobacteria, Deinococcus‐Thermus and Firmicutes likely explained the differences between the two groups (Figure S3). A random forest classifier was established, which achieved modest performance in discriminating tumours from adjacent tissues with an accuracy of 73.2% (Figure S4). Genera belonging to the above‐mentioned four phyla made top contributions to the prediction of tumour vs. adjacent tissues (Figure 2F).

To investigate the functional capabilities of the intratumour microbiota in HCC, we utilised the PICRUSt method to align the 16S rDNA sequences to the relevant genes and pathways. PICRUSt taxonomic functional relationships suggested that the intratumoural microbiome displayed the differential enrichment of environmental information processing, metabolism and cellular processes between tumour and normal tissues (Figure S5).

Finally, we assessed the correlations between taxa in the tumour and normal groups (Figure 2G). We observed clear differences in the structure and complexity of the network between the two groups. In the normal group, positive correlations were detected between ASVs belonging to Firmicutes, Proteobacteria and Deinococcus‐Thermus. In the tumour group, fewer correlations were detected among the populations in the tumours than in those of the adjacent tissues, although few positive correlations existed between Proteobacteria and Actinobacteria.

### 3.4 Interindividual and intratumoural heterogeneity

**FIGURE 3:**
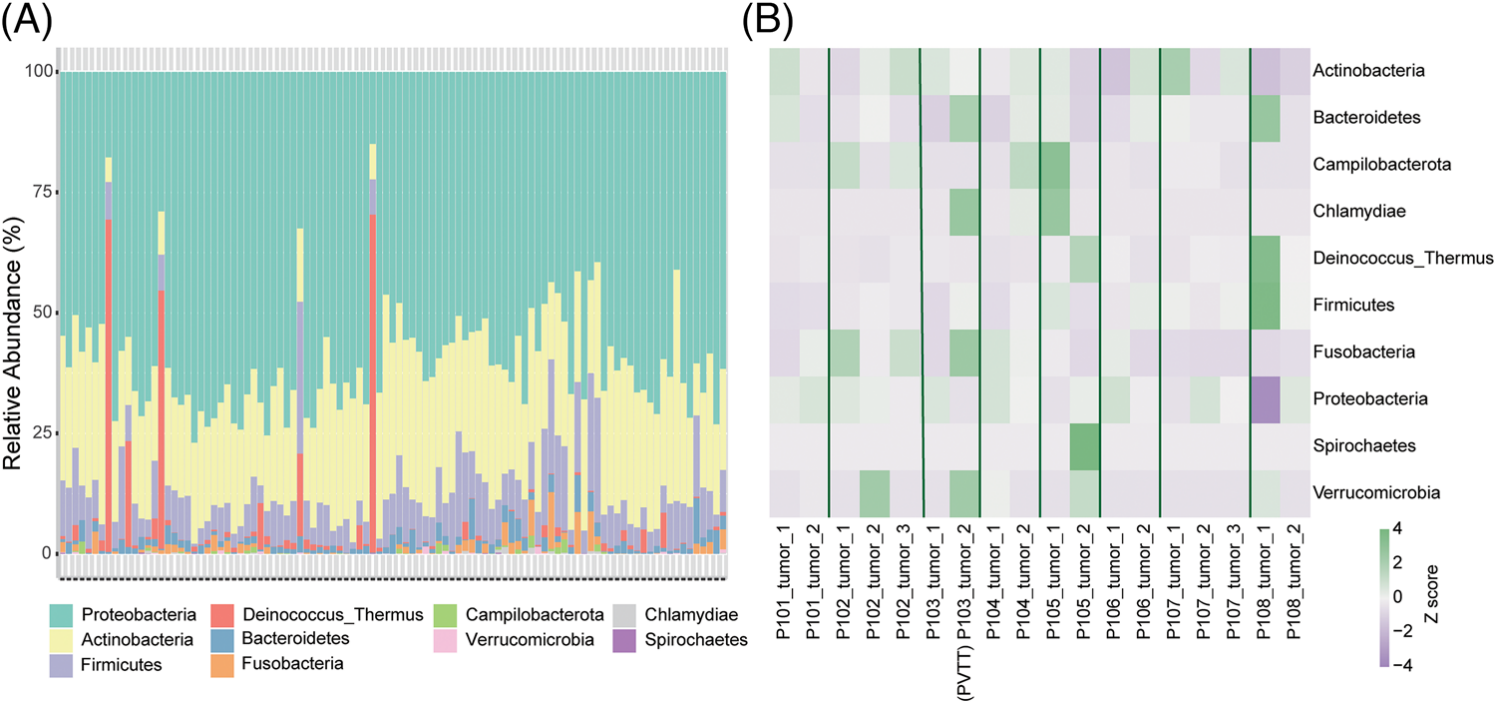
Heterogeneities between individuals or multiple tumour lesions of the microbiome in hepatocellular carcinoma. (A) Stacked column bar graph depicting within‐cohort microbial composition at the phylum level. (B) Heatmap showing the heterogeneity between multiple tumour lesions of patients with multiple tumour samples available. The relative abundance of each phylum is scaled according to the row.

The microbial composition of each individual was plotted at the phylum level. As shown in Figure 3A, the microbial profiles of the patients with HCC showed notable differences. Microbial heterogeneity between multiple tumour lesions was also identified for each individual patient (Figure 3B). Notably, sample P103_tumor_2 was obtained from a tumour thrombus in the portal vein, indicating a change in the microbial profile during the metastasis.

### 3.5 Correlation between intratumoural microbial signatures and clinical characteristics

**FIGURE 4:**
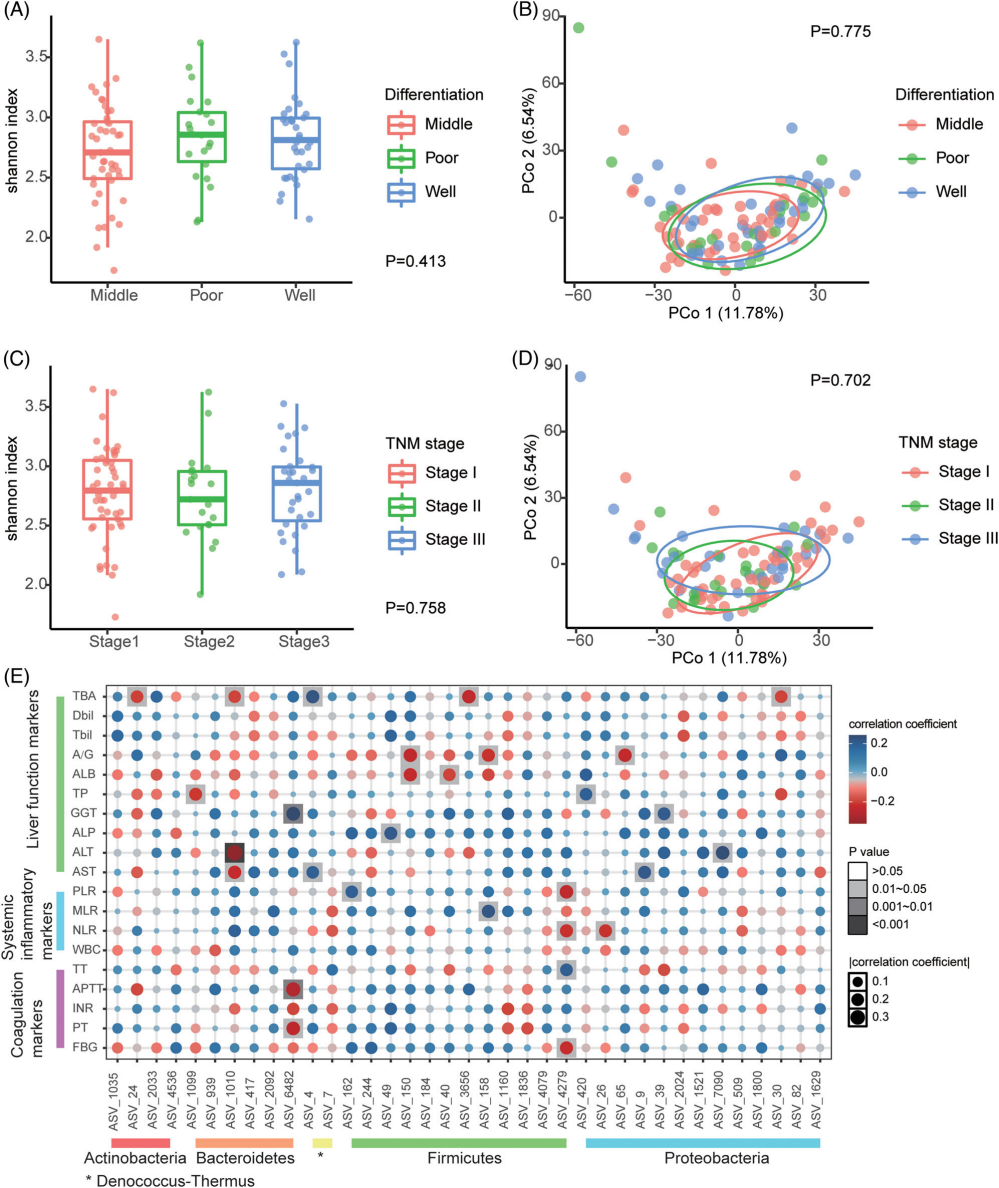
Correlations between the intratumoural microbiome with clinical characteristics. (A–D) The α‐diversity and β‐diversity of tumour tissues categorised by tumour differentiation and TNM stage. (E) Heatmap indicating the correlations between the differential ASVs of tumour (related to Figure 2E) and clinical indices, including liver function tests, coagulation tests and systemic inflammatory markers derived from routine blood tests. A/G: albumin‐globulin ratio; ALB: albumin; ALP: alkaline phosphatase; ALT: alanine aminotransferase; APTT: activated partial thromboplastin time; AST: aspartate aminotransferase; Dbil: direct bilirubin; FBG: fibrinogen; GGT: gamma‐glutamyl transpeptidase; INR: international normalised ratio; MLR: monocyte‐lymphocyte ratio; NLR: neutrophil‐lymphocyte ratio; PLR: platelet‐lymphocyte ratio; PT: prothrombin time; TBA: total bile acid; Tbil: total bilirubin; TP: total protein; TT: thrombin time; WBC: white blood cell.

The relationship between critical clinical characteristics (such as alcohol intake, cirrhosis, HBV infection, tumour differentiation and TNM stage) and microbial profiles was further analysed. The results revealed no significant differences in α‐ and β‐diversities among different tumour differentiation (Figure 4A and B) or TNM stages (Figure 4C and D). Although HBV infection, alcohol intake, type 2 diabetes and being overweight were essential etiological factors of HCC, variation in the diversity and composition of the intratumoural microbiome was hardly observed between the groups (Figures S6–S9). Patients with cirrhosis also showed similar intratumoural microbial diversity and composition as of those without cirrhosis (Figure S10).

The relationship between the differential ASVs mentioned above and biochemical parameters in patients with HCC was further investigated. Actinobacteria were associated with a reduction in total bile acid (TBA) level. Proteobacteria were associated with the elevation of total protein (TP), aspartate aminotransferase (AST), alanine aminotransferase (ALT) and TBA levels, which might indicate a pathophysiological condition of the liver (Figure 4E).

### 3.6 Hepatotypes of intratumoural microbiome

Microbes with antitumour and tumour‐promoting effects might coexist in the TME. Thus, focusing on a single microbe would overlook the comprehensiveness of the microbial signature in the TME. Hence, to comprehensively understand the relationship between the intratumoural microbiome and disease outcome, we identified two clusters as ‘hepatotypes’ of intratumoural microbial profiles.

**FIGURE 5:**
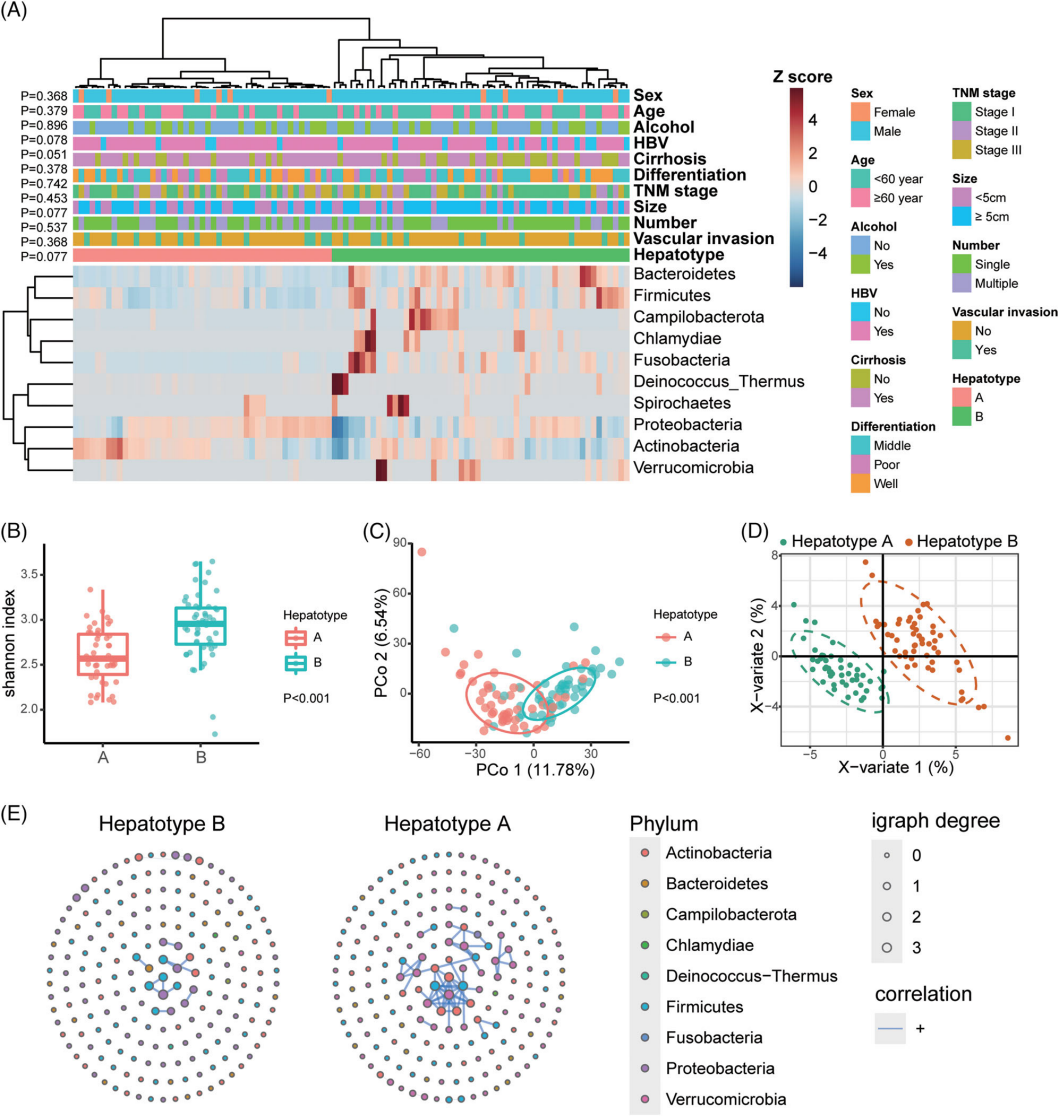
Clustering analysis and characterisation of the microbiome in the two Hepatotypes. (A) Clustering heatmap highlights the microbial signature of hepatotypes A and B, revealing the correlation between the hepatotype and clinical characteristics. The clustering analysis was conducted on the relative abundance of each phylum, which was scaled across all the tumour samples. *Z* scores were calculated and presented in the heatmap. (B) The α‐diversity of tumour samples belonging to Hepatotypes A and B. (C) Principal coordinates analysis (PCoA) based on Manhattan distance of the Hepatotypes A and B. (D) Partial least squares discriminant analysis (PLS‐DA) of the hepatotypes A and B. (E) Microbial networks in hepatotypes A and B. Each dot represents one ASV, and only 200 ASVs with top abundance are shown. The size of each dot represents igraph degree, which reflects the number of significant correlations between the corresponding ASV with the others. Blue lines indicate positive correlations.

The signatures of hepatotypes A and B were classified based on a hierarchical clustering analysis of microbial signatures at the phylum level in the TME of HCC (Figure 5A). Clustering exhibited considerable stability and robustness, as indicated during cross‐validation (Figure S11). Interestingly, multiple lesions from three patients were classified into both hepatotypes A and B, whereas in five other cases with multiple lesions, all lesion samples from each individual were classified either into the hepatotype A or B, but not both. The α‐diversity of hepatotype B was significantly higher than that of hepatotype A (Shannon index, *p* < .001, Figure 5B). Both the PCoA (Manhattan distance, *p* < .001, Figure 5C) and PLS‐DA (Figure 5D) indicated that the β‐diversity of the two hepatotypes differed significantly. Differential analysis using edgeR indicated that 31 ASVs were enriched in hepatotype A and 70 in hepatotype B (Figure S12). LEfSe analysis indicated that Proteobacteria and Actinobacteria showed higher enrichment in hepatotype A, whereas some phyla such as Firmicutes, Fusobacteria and Bacteroidetes showed higher abundance in hepatotype B (Figure S13). The PICRUSt taxonomic functional prediction suggested that hepatotype A exhibited higher enrichment of pathways associated with cancer, cell mortality, and membrane transport (Figure S14).

We further performed a network analysis of the microbial communities of hepatotypes A and B (Figure 5E). We detected different network patterns between the two hepatotypes. Hepatotype A showed complicated positive correlations among microorganisms belonging to the phyla Actinobacteria and Proteobacteria, forming an interactive pattern. However, only a few positive correlations were found in hepatotype B, indicating a relatively scattered pattern of microbial interaction.

### 3.7 Influence of the hepatotype on HCC prognosis

**FIGURE 6:**
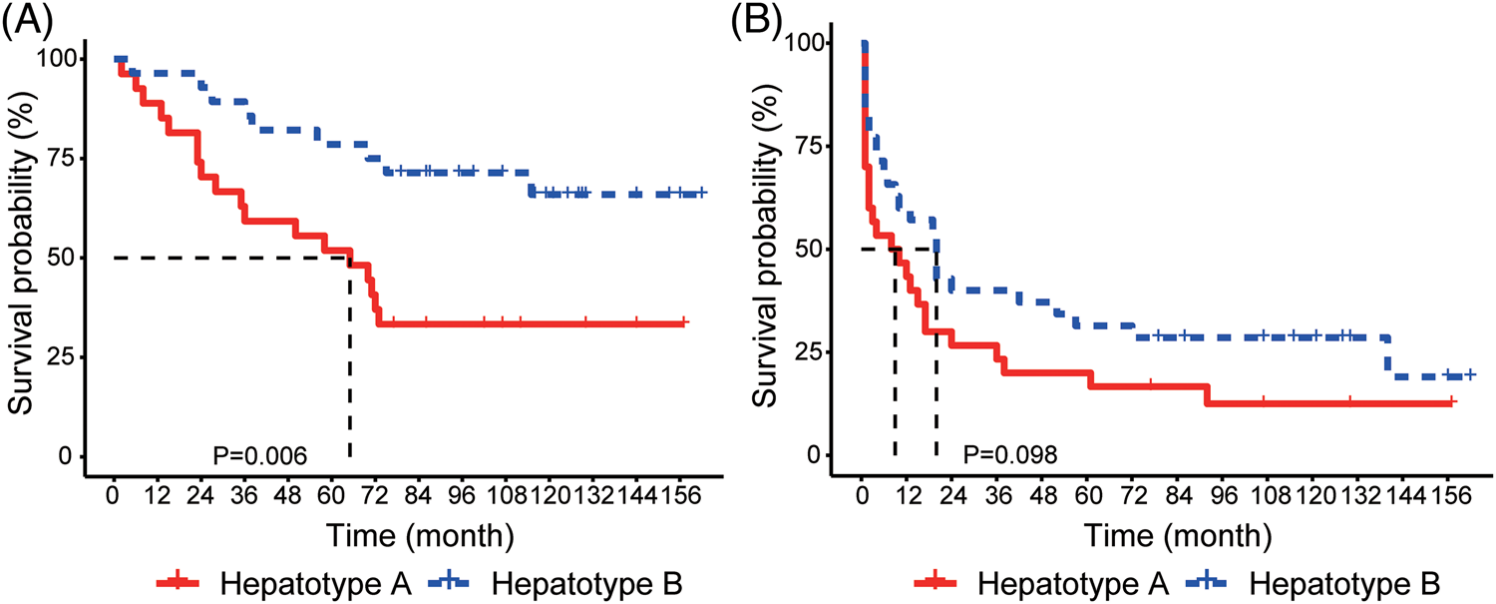
Kaplan–Meier survival curves of patients with hepatocellular carcinoma defined by hepatotypes. (A) Survival curves of overall survival (OS). (B) Survival curves of recurrence‐free survival (RFS).

For three patients whose multiple lesions were classified into different hepatotypes, the patients were deemed hepatotype A, and Kaplan–Meier analysis revealed that hepatotype A had a poor prognosis (Figure 6A and B). The median OS was 65 months in patients classified as hepatotype A, and that for patients categorised into hepatotype B was not reached (*p* = .006). A similar result was obtained for RFS, with a median RFS of eight months for patients with hepatotype A and 20 months for patients with hepatotype B (*p* = .098). Further analysis revealed that the hepatotype was not associated with other clinical characteristics, including TNM stage, alcohol intake, cirrhosis, and HBV infection (Figure 5A and Table S1), suggesting that microbial clustering of hepatotypes could be an independent prognostic factor. Cox regression analysis was applied to demonstrate the predictive ability of the hepatotype along with other important prognostic factors, including tumour differentiation, tumour size, macrovascular invasion and tumour number (Tables S2 and 2). The results validated the predictive power of the hepatotype for both OS (HR = 0.296, 95% CI: 0.126−0.699, *p* = .005, Table 2) and RFS (HR = 0.504, 95% CI: 0.276−0.918, *p* = .025, Table 2).

**TABLE 2:** Multivariate Cox regression analysis for survival prediction.

	Overall survival		Multivariate analysis	
	HR (95% CI)	*p*	HR (95% CI)	*p*
Hepatotype				
A	Reference		Reference	
B	0.296 (0.126−0.699)	.005	0.504 (0.276−0.918)	.025
Differentiation				
Well	Reference		Reference	
Medium	0.770 (0.299−1.980)	.588	0.989 (0.519−1.885)	.974
Poor	2.167 (0.776−6.050)	.140	1.199 (0.542−2.652)	.653
Tumour size				
<5 cm	Reference		Reference	
≥5 cm	0.819 (0.362−1.852)	.631	0.761 (0.419−1.383)	.370
Macrovascular invasion				
No	Reference		Reference	
Yes	1.288 (0.453−3.666)	.635	2.841 (1.426−5.662)	.003
Tumour number				
Single	Reference		Reference	
Multiple	0.620 (0.262−1.465)	.276	1.148 (0.632−2.087)	.650
Cirrhosis				
No	Reference		Reference	
Yes	6.108 (1.803−20.696)	.004	1.938 (0.973−3.860)	.060

To demonstrate which microorganisms might play a role in the differences of prognosis, we identified the top 20 genera that discriminate between hepatotype A and hepatotype B using a random forest model (Figure S15A). The random forest classifier achieved an accuracy of 86.1% at the genus level (Figure S15B). Patients were then categorised into groups based on the relative abundance of the 20 genera. *Methylobacterium* and *Akkermansia* were significant prognostic markers for both OS and RFS (Figure S16). Notably, *Methylobacterium* was significantly enriched in hepatotype B (Figures S13 and S16F). Similarly, the relative abundance of *Akkermansia* tended to be higher in hepatotype B than in hepatotype A, just bordering on statistical significance (*p* = .061, Figure S16C). The results corresponded with the finding that low levels of *Akkermansi*a or *Methylobacterium* were associated with poor long‐term outcomes.

## 4 DISCUSSIONS

Accumulating evidence has demonstrated the presence and potential roles of microbiota in the TME. The microbiota that resides within tumours is tumour‐specific, and mechanistic studies illustrate that tumour‐associated microbiota can influence cancer initiation, progression, and responses to chemotherapy or immunotherapy.^9^, ^10^ The presence and profile of a microbiome in HCC TME has been established by recent studies; however, its clinical significance in relation to HCC is not well elucidated.

In this study, we explored the microbiota in the HCC TME based on a large‐scale analysis of samples from tumour and adjacent normal tissues of 91 patients with HCC. We proved the presence of an intratumoural microbiome using FISH, and characterised the microbial profiles in tumourous and adjacent tissues using 16S rDNA sequencing. Further analysis revealed a distinct microbial pattern between the tumour and adjacent tissues of HCC, indicating an independent microbiome signature for HCC. Similar to that reported by previous studies on intratumoural microbiota, we identified differences in the abundance of specific taxa between tumours and adjacent normal tissues. The results have revealed that the tumour‐associated microbiota of HCC primarily consisted of Actinobacteria, Proteobacteria and Firmicutes, the abundance of which varied significantly between the specimens. Moreover, we presented the heterogeneity of the microbiome in HCC among a subset of patients with multiple lesions. Furthermore, the relative abundance of taxa at the phylum level was clustered into HCC hepatotypes by integrating the overall microbial signature of the tumour samples. The results demonstrated that the hepatotype carried a significant prognostic value when adjusted for previous common prognostic factors such as tumour differentiation, tumour size, tumour number and macrovascular invasion. High levels of *Akkermansia* and *Methylobacterium* were predictive of favourable OS and RFS.

Compared with recent researches,^14^, ^15^, ^16^, ^17^, ^18^, ^19^, ^20^ our study presents new findings and has potential translational value. On one hand, with a substantial sample size, we revealed the prognostic value of intratumoural microbiota in HCC. While the proposed hepatotype based on intratumoural microbiome requires validation in future studies, our findings could enlighten studies on the potential clinical translation of intratumoural microbiome of HCC. On the other hand, the heterogeneity of intratumoural microbiome in HCC was demonstrated in our study. Both interpatient heterogeneity and intratumoural heterogeneity were present. Two recent studies that also identified the intratumoural microbiome and assessed its heterogeneity suggested that the nonrandom distribution of the microbiota in tumours correlated with carcinogenesis or cancer progression.^30^, ^31^ Whether the heterogeneity of the intratumoural microbiome in HCC impacts tumour development and patient clinical outcomes warrants further investigation.

Previous studies have proposed that the intratumoural microbiome interacts with the host through three primary mechanisms^32^: (1) direct impact on tumourigenesis through DNA damage, (2) regulation of oncogenic signalling pathways and (3) modulation of the host's immune response and inflammation. However, it remains unclear how the intratumoural microbiome influences HCC prognosis. Upon further examination of the hepatotypes A and B, we noticed that hepatotype A, which was associated with a poorer prognosis, was characterised by lower α‐diversity and an abundance of Proteobacteria and Actinobacteria species. These findings were consistent with that of previous studies that have demonstrated the presence of Proteobacteria in HCC.^14^ Evidence indicates that Gram‐negative bacteria, specifically Proteobacteria, are involved in the pathogenesis of endotoxemia (also known as metabolic endotoxemia)^33^, ^34^ and inflammation.^34^, ^35^ Similar to Proteobacteria, Actinobacteria enrichment has been previously detected in tumour tissue and gut microbiota of patients with poor prognosis, indicating a similar role.^36^, ^37^

Proteobacteria has been widely detected in the gastrointestinal tract microbiota of patients with HCC. According to previous studies, an increase in Proteobacteria abundance in the gut may shorten patient lifespan on onset of cancer and increase the risk of cachexia.^38^, ^39^ Considering that the gut–liver axis plays a crucial role in the pathogenesis of liver diseases and that most gut bacteria can colonise multiple tumourous environments,^40^, ^41^ bacterial translocation from the gut to the liver may also occur.

The hepatotype A also had lower levels of *Akkermansia* and *Methylobacterium*. *Akkermansia muciniphila* is a typical species of the genus *Akkermansia*. Numerous studies have demonstrated that *Akkermansia muciniphila* is a commensal probiotic. Studies have uncovered that a decrease in the levels of *Akkermansia muciniphila* is associated with the progression of cancer and a poor response to cancer immunotherapies.^42^ Our study found that high levels of *Akkermansia* in HCC tissues were associated with a favourable prognosis, suggesting that *Akkermansia* may also play a beneficial role in the HCC TME. A recent study indicated that *Methylobacterium* within tumours was associated with worse outcomes in patients with gastric cancer,^43^ while our study found the opposite. However, organisms may have varying roles in different anatomical sites or pathophysiological conditions. Future researches are warranted to explore how the key microorganisms in TME influence HCC prognosis.

Network analysis and comparisons were made between the tumour and normal groups and the different hepatotypes. Notably, the network complexity in the tumours of patients with HCC was significantly lower than that in the adjacent normal tissues. Previous research on the stability of microbial communities has demonstrated that the modularity of microbial networks continues to decrease under persistent stress.^44^ Overall, the disease burden may impede the compartmentalisation of microbial associations and create communities dominated by tumour‐friendly species, which could destabilise microbiomes and lead to dysbiosis in certain areas. Dysbiosis of intratumoural microorganisms may impact the TME, thereby promoting tumour progression.

One of the limiting factors of this study is its cross‐sectional design, which prevented us from elucidating the longitudinal perspective of relevance. Another factor is the lack of external validation; therefore, the predictive value of the hepatotypes in patients with HCC needs to be verified in other independent cohorts. Researchers should exercise caution in generalising the findings of our study, particularly in cohorts with different baseline characteristics compared to ours. Besides, despite using 16S rDNA sequencing to describe the intratumoural microbiome of patients with HCC and its impact on clinical outcomes after surgery, we were unable to provide insights into the potential mechanisms underlying the role of the intratumoural microbiome in HCC. Further investigations into biological mechanisms are of great significance as they can help uncover the impacts of the intratumoural microbiome in HCC and identify key links for clinical intervention. Finally, this study is limited by its retrospective nature and lack of healthy participants for comparison. Currently, recent studies have confirmed the presence of the microbiome within tumours via immunohistochemical staining and FISH; therefore, further researches need to extend beyond establishing correlations and explore causality. Nevertheless, our findings adequately illustrate the microbial characteristics in the HCC TME and the bacteria‐associated signatures that contribute to tumour development in various prognoses. Comprehending the dynamics of intratumoural microbiota during liver carcinogenesis and its development can yield new prognostic biomarker discoveries and enhance HCC prognosis.

## 5 CONCLUSIONS

In conclusion, we characterised the intratumoural microbiome in the HCC TME and discovered that the pattern of the intratumoural microbiome, defined as the hepatotype, significantly affected the survival of patients with HCC after surgery. Thus, the intratumoural microbiome can help predict patient outcomes and is unique to each hepatotype – one that indicates a favourable prognosis may contribute to forming a favourable TME and favourable patient outcome.

## CONFLICT OF INTEREST STATEMENT

The authors declare no competing interests.

## Supporting information

Supporting Information

## Data Availability

Data relevant to the study have been included in this article or uploaded as supplementary materials. The raw sequencing data can be accessed from the National Genomics Data Center (NGDC) under accession number HRA004424 (https://ngdc.cncb.ac.cn/gsa‐human/browse/HRA004424). Related data can also be acquired from the corresponding authors on reasonable request.
