# Supplementary material for: Intratumoural microbiome can predict the prognosis of hepatocellular carcinoma after surgery
Source: Clin Transl Med. 2023 Jul 18;13(7):e1331. doi: 10.1002/ctm2.1331 (PMC10353526; doi:10.1002/ctm2.1331)
Supplement: Supplementary file 1 — Supporting Information [file CTM2-13-e1331-s001.docx]

**Intratumoral microbiome can predict the prognosis of hepatocellular carcinoma after surgery**

Lejia Sun^1,2,3,4#^, Xindi Ke^1#^, Ai Guan^5#^, Bao Jin^1#^, Jiangming Qu^5^, Yinhan Wang^5^, Xiang Xu^5^, Changcan Li^1^, Hang Sun^1^, Hengyi Xu^5^, Gang Xu^6,7^, Xinting Sang^1^, Yifei Feng^2,3,4*^, Yueming Sun^2,3,4*^, Huayu Yang^1*^, Yilei Mao^1*^

1. Department of Liver Surgery, Peking Union Medical College (PUMC) Hospital, PUMC & Chinese Academy of Medical Sciences, Beijing, China.

2. Department of General Surgery, The First Affiliated Hospital of Nanjing Medical University, Nanjing, China.

3. The First School of Clinical Medicine, Nanjing Medical University, Nanjing, China.

4. Colorectal Institute of Nanjing Medical University, Nanjing, China.

5. Peking Union Medical College (PUMC) & Chinese Academy of Medical Sciences, Beijing, China.

6. Liver Transplant Center, Organ Transplant Center, West China Hospital of Sichuan University, Chengdu 610041, China.

7. Laboratory of Liver Transplantation, Key Laboratory of Transplant Engineering and Immunology, NHC, West China Hospital of Sichuan University, Chengdu 610041, China

# Lejia Sun, Xindi Ke, Ai Guan, and Bao Jin contributed equally to this study and share co-first authorship.

* Corresponding authors: Yilei Mao, 0000-0003-0449-4223, pumch-liver@hotmail.com; Huayu Yang, 0000-0001-9791-3559, [dolphinyahy@hotmail.com](mailto:dolphinyahy@hotmail.com); Yueming Sun, 0000-0001-8641-1668, [sunyueming@njmu.edu.cn](mailto:sunyueming@njmu.edu.cn); Yifeng Feng, 0000-0002-7946-446X, [fengyifei1982@163.com](mailto:fengyifei1982@163.com).

Supplementary Table 1…………………………………………………………………………... …4

Supplementary Table 2……………………………………………………………………………...5

Supplementary Figure 1…………………………………………………………………....….…….6

Supplementary Figure 2……………………………………………………….……….……………7

Supplementary Figure 3…………………………………………………………………….……….8

Supplementary Figure 4…………………………………………………………………….……….9

Supplementary Figure 5……………………………………………………………….………...…10

Supplementary Figure 6…………………………………………………………………….……...11

Supplementary Figure 7……………………………………………………………………………12

Supplementary Figure 8……………………………………………………………………………13

Supplementary Figure 9……………………………………………………………………………14

Supplementary Figure 10……………………………………………………………………….….15

Supplementary Figure 11……………………………………………………………………….….16

Supplementary Figure 12……………………………………………………………………….….17

Supplementary Figure 13……………………………………………………………………….….18

Supplementary Figure 14……………………………………………………………………….….20

Supplementary Figure 15…………………………………………………………….…………….21

Supplementary Figure 16…………………………………………………………….…………….22

**Supplementary Table 1.** Baseline characteristics of patients classified into hepatotypes A and B.

| **Characteristics** | **Hepatotype A**  **(n=43)** | **Hepatotype B**  **(n=48)** | **P** |
| --- | --- | --- | --- |
| **Age** (≥year) | 16 (37.2%) | 21 (43.8%) | 0.526 |
| **Gender** (male) | 39 (90.7%) | 44 (91.7%) | 0.579 |
| **Alcohol intake** (Yes) | 17 (39.5%) | 17 (35.4%) | 0.685 |
| **HBV infection** (Yes) | 38 (88.4%) | 35 (72.9%) | 0.065 |
| **Cirrhosis** (Yes) | 33 (76.7%) | 29 (60.4%) | 0.095 |
| **Tumor differentiation** |  |  | 0.451 |
| Well | 16 (37.2%) | 12 (25.0%) |  |
| Medium | 19 (44.2%) | 25 (52.1%) |  |
| Poor | 8 (18.6%) | 11 (22.9%) |  |
| **TNM stage** |  |  | 0.855 |
| I | 21 (48.8%) | 24 (50.0%) |  |
| II | 10 (23.3%) | 9 (18.8%) |  |
| III | 12 (27.9%) | 15 (31.2%) |  |
| **Tumor size** (>5cm) | 23 (53.5%) | 30 (62.5%) | 0.384 |
| **Tumor number** (multiple) | 17 (39.5%) | 14 (29.2%) | 0.297 |
| **Macrovascular invasion** (Yes) | 10 (23.3%) | 10 (20.8%) | 0.781 |

Notes: Supplementary Table 1 was related to Figure 5A. For three patients whose multiple lesions were classified into different hepatotypes, the patients were regarded as hepatotype A. The chi-square tests or Fisher’s exact tests were applied as appropriate.

**Supplementary Table 2.** Univariate Cox regression analysis for survival prediction.

|  | **Overall survival** | | **Recurrence-free survival** | |
| --- | --- | --- | --- | --- |
|  | **HR (95% CI)** | **P** | **HR (95% CI)** | **P** |
| **Hepatotype** |  |  |  |  |
| A | reference |  | reference |  |
| B | 0.339 (0.151–0.759) | 0.008 | 0.646 (0.374–1.117) | 0.117 |
| **Differentiation** |  |  |  |  |
| Well | reference |  | reference |  |
| Medium | 0.553 (0.229–1.338) | 0.189 | 0.747 (0.403–1.385) | 0.354 |
| Poor | 1.321 (0.490–3.563) | 0.583 | 1.102 (0.519–2.338) | 0.801 |
| **Tumor size** |  |  |  |  |
| <5cm | reference |  | reference |  |
| ≥5cm | 0.854 (0.400–1.822) | 0.683 | 0.973 (0.559–1.694) | 0.924 |
| **Macrovascular invasio**n |  |  |  |  |
| No | reference |  | reference |  |
| Yes | 1.157 (0.466–2.870) | 0.753 | 2.528 (1.365–4.681) | 0.003 |
| **Tumor number** |  |  |  |  |
| Single | reference |  | reference |  |
| Multiple | 0.811 (0.364–1.806) | 0.609 | 1.153 (0.658–2.018) | 0.619 |
| **Cirrhosis** |  |  |  |  |
| No | reference |  | reference |  |
| Yes | 5.514 (1.655–18.377) | 0.005 | 1.984 (1.037–3.794) | 0.038 |

**Supplementary Figure 1.** 16S rDNA sequencing was conducted in three previously collected pairs of tumor and adjacent tissues as well as three pairs of samples that were prospectively collected. (A) The α-diversity of the prospectively and retrospectively collected tumor and adjacent normal tissues. (B) The β-diversity of the prospective and retrospective samples visualized via principal component analysis. (C) Main composition of the prospective and retrospective groups at the phylum level. No significant difference was observed between the two groups. (D) Volcano plot illustrating the differential amplicon sequence variants (ASVs) between prospectively and retrospectively collected samples. ASVs with a relative abundance higher than 0.05% were presented in the volcano plot.

**Supplementary Figure 2.** Comparisons of the 10 phyla in tumor and adjacent normal tissues. Mann–Whitney U-test was applied in comparisons of relative abundance between the two groups. Different letters above the boxplots, namely a and b, indicate significant differences.

**Supplementary Figure 3.** Linear discriminant analysis effect size (LEfSe) analysis, depicting the differential taxa between the microbiome of hepatocellular carcinoma tumor and adjacent normal tissues. (A) Taxonomic cladogram of the LEfSe analysis. Each node represents a specific taxon (p, phylum; c, class; o, order; f, family; g, genus). Yellow nodes denote the taxonomic features that are not significantly differentiated between tumor and normal tissues. Green nodes denote the taxonomic types with more abundance in tumors than in normal tissues, while red nodes represent the taxonomic types more abundant in normal tissues. (B) Histogram of linear discriminant analysis (LDA) score of taxa with differential abundance between tumor and normal tissues. Only features with LDA score (log10) > 3.0 and P < 0.05 are shown.

**Supplementary Figure 4.** Five-fold cross validation of the random forest model to discriminate tumor from adjacent tissues. Random forest models were developed at different taxonomic levels with the genus level presents the best performance (data not shown). When the predictive model was performed and validated at the genus level, the inclusion of 20 genera achieved the least error rate (error rate 26.8%, accuracy 73.2%). The 20 genera which make top contributions to the classifier are listed in Figure 2F.

**Supplementary Figure 5.** Bar plot of the differentially functional pathways between tumor and normal tissues. Comparisons were conducted via Welch’s t-test, and only pathways with Benjamini-Hochberg false discovery rate < 0.05 are shown.

**Supplementary Figure 6.** Microbial diversity and composition in tumor samples of patients with and without HBV infection. (A) Boxplot of Shannon index. (B) Principal co-ordinates analysis (PCoA) based on Manhattan distance. (C–D) Pie plots of microbial composition at the phylum level in patients without HBV infection (C) and with HBV infection (D).

**Supplementary Figure 7.** Microbial diversity and composition in tumor samples of patients with and without alcohol intake. (A) Boxplot of Shannon index. (B) Principal co-ordinates analysis (PCoA) based on Manhattan distance. (C–D) Pie plots of microbial composition at the phylum level in patients without alcohol intake (C) and with alcohol intake (D).

**Supplementary Figure 8.** Microbial diversity and composition in tumor samples of patients with and without type 2 diabetes. (A) Boxplot of Shannon index. (B) Principal co-ordinates analysis (PCoA) based on Manhattan distance. (C–D) Pie plots of microbial composition at the phylum level in patients with diabetes (C) and without diabetes (D).

**Supplementary Figure 9.** Microbial diversity and composition in tumor samples of overweight and non-overweight patients. (A) Boxplot of Shannon index. (B) Principal co-ordinates analysis (PCoA) based on Manhattan distance. (C–D) Pie plots of microbial composition at the phylum level in overweight patients (C) and non-overweight patients (D).

**Supplementary Figure 10.** Microbial diversity and composition in tumor samples of patients with and without cirrhosis. (A) Boxplot of Shannon index. (B) Principal co-ordinates analysis (PCoA) based on Manhattan distance. (C–D) Pie plots of microbial composition at the phylum level in patients without cirrhosis (C) and with cirrhosis (D).

**Supplementary Figure 11.** Boxplot for the cross-validation of the clustering analysis (related to Figure 5A). Certain percentage of the tumor samples were randomly extracted for 20 times respectively, and the accuracy of the clustering analysis was calculated.

**Supplementary Figure 12.** Volcano plot of differential amplicon sequence variants (ASVs) between Hepatotypes A and B. ASVs significantly depleted in Hepatotype A are shown in green; those significantly enriched in Hepatotype A are shown in red (|log2 fold change| > 1, P < 0.05, and false discovery rate < 0.2).

**Supplementary Figure 13.** Linear discriminant analysis effect size (LEfSe) analysis, depicting the differential taxa between the microbiome of Hepatotypes A and B. (A) Taxonomic cladogram of the LEfSe analysis. Each node represents a specific taxon (p, phylum; c, class; o, order; f, family; g, genus). Yellow nodes denote the taxonomic features that are not significantly differentiated between Hepatotypes A and B. Red nodes denote the taxonomic types with more abundance in Hepatotype A, while green nodes represent the taxonomic types more abundant in Hepatotype B. (B) Histogram of linear discriminant analysis (LDA) score of taxa with differential abundance between Hepatotypes A and B. Only features with LDA score (log 10) > 3.0 and P < 0.05 are shown.

**Supplementary Figure 14.** Bar plot of the differentially functional pathways between Hepatotypes A and B. Comparisons were conducted via Welch’s t-test, and only pathways with Benjamini-Hochberg false discovery rate < 0.05 are shown.

**Supplementary Figure 15.** Random forest model to discriminate hepatotypes A and B, and its five-fold cross validation. (A) Bar plot of 20 genera with the top ability to discriminate hepatotypes A and B computed from a random forest model. The phyla are indicated by different colors. (B) Five-fold cross validation of the random forest model. Inclusion of the 20 genera achieved an accuracy of 86.1% and error rate of 13.9%.

**Supplementary Figure 16.** Prognostic value of *Akkermansia* and *Methylobacterium* for hepatocellular carcinoma (HCC), and their relative abundance in hepatotypes A and B. (A–B) Survival curves of patients with HCC stratified by levels of Akkermansia. (C) Relative abundance of *Akkermansia* in hepatotypes A and B. (D–E) Survival curves of patients with HCC stratified by levels of *Methylobacterium*. (F) Relative abundance of Methylobacterium in hepatotypes A and B. Mann–Whitney U-test was applied in comparisons of relative abundance between groups. The cutoff values for survival analyses were determined according to the medium values of relative abundance.
